# Analysis of Genetic Variants Associated with Levels of Immune Modulating Proteins for Impact on Alzheimer’s Disease Risk Reveal a Potential Role for SIGLEC14

**DOI:** 10.3390/genes12071008

**Published:** 2021-06-30

**Authors:** Benjamin C. Shaw, Yuriko Katsumata, James F. Simpson, David W. Fardo, Steven Estus

**Affiliations:** 1 Department of Physiology, University of Kentucky, Lexington, KY 40506, USA; benjamin.shaw@uky.edu (B.C.S.); jfsimp01@uky.edu (J.F.S.); 2 Sanders-Brown Center on Aging, University of Kentucky, Lexington, KY 40506, USA; david.fardo@uky.edu; 3 Department of Biostatistics, University of Kentucky, Lexington, KY 40506, USA; katsumata.yuriko@uky.edu

**Keywords:** ITIM, ITAM, *SIGLEC14*, *SIGLEC5*, copy number variation, CNV, GWAS

## Abstract

Genome-wide association studies (GWAS) have identified immune-related genes as risk factors for Alzheimer’s disease (AD), including *TREM2* and *CD33*, frequently passing a stringent false-discovery rate. These genes either encode or signal through immunomodulatory tyrosine-phosphorylated inhibitory motifs (ITIMs) or activation motifs (ITAMs) and govern processes critical to AD pathology, such as inflammation and amyloid phagocytosis. To investigate whether additional ITIM and ITAM-containing family members may contribute to AD risk and be overlooked due to the stringent multiple testing in GWAS, we combined protein quantitative trait loci (pQTL) data from a recent plasma proteomics study with AD associations in a recent GWAS. We found that pQTLs for genes encoding ITIM/ITAM family members were more frequently associated with AD than those for non-ITIM/ITAM genes. Further testing of one family member, *SIGLEC14* which encodes an ITAM, uncovered substantial copy number variations, identified an SNP as a proxy for gene deletion, and found that gene expression correlates significantly with gene deletion. We also found that *SIGLEC14* deletion increases the expression of *SIGLEC5*, an ITIM. We conclude that many genes in this ITIM/ITAM family likely impact AD risk, and that complex genetics including copy number variation, opposing function of encoded proteins, and coupled gene expression may mask these AD risk associations at the genome-wide level.

## 1. Introduction

Genome-wide association studies (GWAS) have identified a set of polymorphisms that modulate the risk of Alzheimer’s disease (AD) [1,2,3,4,5,6]. The pathways implicated in this process include innate immunity, cholesterol homeostasis, and protein trafficking [7,8,9]. Four of these genes, *TREM2*, *CD33*, *PILRA,* and *FCER1G*, are members of the family of non-catalytic tyrosine-phosphorylated receptors (NTRs), which function through immunomodulatory tyrosine-phosphorylated activating motifs (ITAMs) or inhibitory motifs (ITIMs). The underlying immunomodulatory pathway is further implicated by AD-associated variants in phospholipase C (*PLCG2*) and *INPP5D* which encode proteins acting downstream of these ITAM- and ITIM-containing proteins. Functional studies have informed the current hypothesis that the variants associated with AD in the ITAM/ITIM family modulate inflammation and phagocytosis [10,11,12,13,14,15,16,17,18].

The ITAM family, including *TREM2*, recruit kinases such as spleen tyrosine kinase (Syk) and phosphoinositide 3-kinase (PI3K) to induce downstream signaling, while the ITIM family, including *CD33*, recruit phosphatases such as SHP-1 to dephosphorylate Syk and ITAMs, thereby counteracting ITAM activity [19]. These ITAM and ITIM proteins are predominantly expressed in immune cells such as microglia. Overall, these and other studies have shown that microglia contribute to AD pathogenesis, a concept that has been reviewed recently [20,21,22].

The critical barrier to progress in translating GWAS candidate genes to treatments is elucidating the actions of the functional variant at the molecular level, i.e., splicing (sQTL), gene expression (eQTL), or protein level (pQTL), to understand whether the pathway affected is detrimental or beneficial to disease risk. GWAS single nucleotide polymorphisms (SNPs) in AD are frequently identified as eQTLs in the brain [23]. Sun et al. have used GWAS to identify pQTLs for the plasma proteome, including ITIM and ITAM-containing proteins [24]. To investigate the hypothesis that these pQTLs may uncover additional AD-related genes that may have been overlooked in AD GWAS because of their stringent false-discovery rate controls, we examined the Sun et al. cis-pQTL data together with the Jansen et al. AD GWAS results. Parsing the proteins from the genome-wide significant cis-pQTL dataset by whether or not an ITIM/ITAM domain was present, and then examining whether the associated SNP is nominally significant (*p* < 0.05) for AD association, found a significant overrepresentation of ITIM/ITAM encoding genes with nominal AD associations. Since one of these genes, *SIGLEC14*, has been reported to be deleted in some individuals, we investigated further and found that the pQTL and AD SNP, rs1106476, is a proxy for the previously identified deletion polymorphism [25]. We defined this deletion further by identifying additional *SIGLEC14* copy number variants and by determining the effect of *SIGLEC14* copy number on the expression of *SIGLEC14* and the neighboring *SIGLEC5*. We conclude that variants in ITIM/ITAM family members, including *SIGLEC14*, represent underappreciated potential genetic risk factors for AD.

## 2. Materials and Methods

### 2.1. Preparation of gDNA, RNA, and cDNA from Human Tissue

Human blood and anterior cingulate autopsy tissue from 61 donors were generously provided by the Sanders-Brown Alzheimer’s disease center neuropathology core and have been described elsewhere [26]. The matched brain and blood samples were from deceased individuals with an average age at death of 82.4 ± 8.7 (mean ± SD) years for non-AD and 81.7 ± 6.2 years for AD subjects. The average postmortem interval (PMI) for non-AD and AD subjects was 2.8 ± 0.8 and 3.4 ± 0.6 h, respectively. Non-AD and AD samples were comprised of 48% and 55% female subjects. MMSE scores were, on average, 28.4 ± 1.6 for non-AD subjects and 11.9 ± 8.0 for AD subjects. These samples were used for genotyping and gene expression studies. Three additional blood samples matched to whole-genome sequencing (WGS) data were obtained to confirm WGS observations of additional *SIGLEC14* copies. DNA from these patients was prepared using a QIAamp DNA Blood Mini kit (Qiagen, Germantown, MD, USA) per the manufacturer’s instructions.

### 2.2. Genotyping and Copy Number Variant Assays

Copy number variation in *SIGLEC14* was determined using a TaqMan-based copy number variant (CNV) assay (Invitrogen, Waltham, MA, USA; Catalog number 4400291, Assay number Hs03319513_cn) compared to *RNAse P* (Invitrogen, 4403326). Amplification and quantitation were performed per manufacturer instructions. Genotyping the rs1106476 was performed with a custom TaqMan assay (Invitrogen). This assay discriminates rs1106476 and rs872629, which are in perfect LD. As coinherited SNPs, this variant is also known as rs35495434.

### 2.3. Gene Expression by qPCR

Gene expression was quantified by qPCR with PerfeCTa SYBR Green master mix as previously described [14]. *SIGLEC14* was quantified with primers corresponding to a sequence in exons 3 and 5: 5′-CAGGTGAAACGCCAAGGAG-3′ and 5′-GCGAGGAACAGGGACTGG-3′. *SIGLEC5* was quantified with primers corresponding to sequences in exons 4 and 5: 5′-ACCATCTTCAGGAACGGCAT-3′ and 5′-GGGAGCATCACAGAGCAGC-3′. Cycling conditions for all qPCRs were as follows: 95 °C, 2 min; 95 °C, 15 s, 60 °C, 15 s, 72 °C, 30 s, 40 cycles. Copy numbers present in the cDNA were determined relative to standard curves that were executed in parallel [19].

### 2.4. WGS Data Analysis

To investigate the frequency and range of *SIGLEC14* CNV, we performed a read-depth analysis for WGS data. We obtained compressed sequence alignment map (CRAM) files from the AD sequencing project (ADSP) and AD Neuroimaging (ADNI). We extracted paired-end reads mapped to the *SIGLEC14-SIGLEC5* locus under Genome Reference Consortium Human Build 38 (GRCh38/hg38), and then computed the depth at each position using the samtools depth function [27].

### 2.5. Statistical Analyses

The association of cis-pQTL proteins containing ITIM/ITAM domains and AD-associated SNPs was calculated using a simple chi-square test. Gene expression was analyzed by using JMP14 Pro using one-way analysis of variance (ANOVA) followed by Tukey’s post-hoc multiple testing correction and graphed in GraphPad Prism 8.

## 3. Results

### 3.1. ITIM/ITAM pQTLs Are Overrepresented in AD GWAS Results

To evaluate whether pQTLs for ITIM or ITAM-containing proteins were associated with AD, we compiled a list of ITIM and ITAM-containing proteins from prior reviews [28,29,30,31]. The resulting list contained 187 genes and is provided as Supplemental Table S1. The cis-acting pQTLs from Sun et al. and AD associations from Jansen et al. were then matched by chromosomal coordinates [2,24]. Both datasets were provided under Genome Reference Consortium Human Build 37 (GRCh37/hg19). Genes were then subset as either coding for an ITIM/ITAM gene or not and nominally significant (*p* < 0.05) for AD association or not. The SNPs which are associated with both ITIM/ITAM protein levels in plasma and AD risk are shown in Table 1. We found that pQTLs that affect ITIM or ITAM genes were significantly overrepresented in nominally significant AD associations (*p* = 6.51 × 10^−5^, $\chi_{1}^{2}$ = 15.95, Table 2).

### 3.2. SIGLEC14 pQTL Is a Proxy for the Deletion Polymorphism

**Figure 1:**
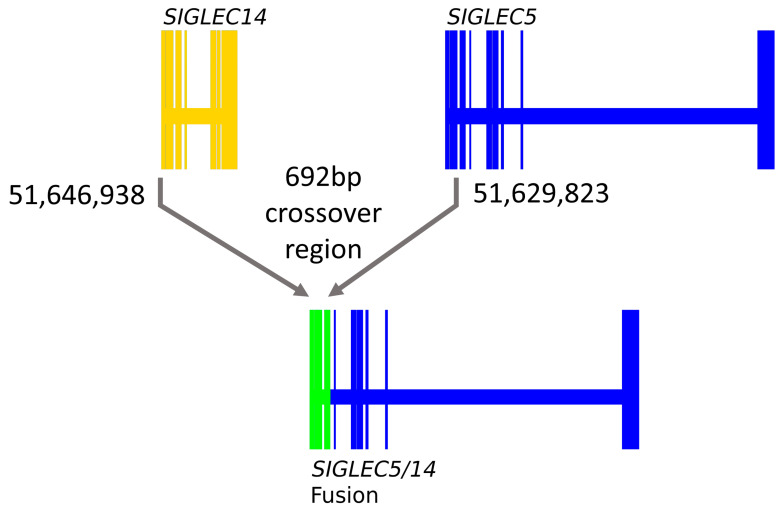
Identification of the *SIGLEC14* deletion site. Coordinates in both are for reference genome. Exons 1-3 of *SIGLEC14* and *SIGLEC5* are identical which confounds exact determination of the crossover event. The yellow region depicts *SIGLEC14*, the blue region depicts *SIGLEC5*, while the green region depicts the 692 bp region of complete identity where the crossover deletion occurs.

Previous reports have identified a *SIGLEC14* deletion [25]. Given the strong pQTL signal from rs1106476 on SIGLEC14 reported by Sun et al., and the fact that rs1106476 is within the neighboring *SIGLEC5* gene, yet has a cis-pQTL effect on SIGLEC14, we hypothesized that rs1106476 is a proxy for the *SIGLEC14* deletion polymorphism. To test this hypothesis, we genotyped a set of DNA samples for rs1106476 and quantified genomic copy number variation (CNV). We found that the proxy SNP correlates with *SIGLEC14* deletion well but not perfectly (*p* < 0.0001, $\chi_{2}^{2}$ = 38.40) (Table 3). To better understand this deletion, we then sequenced the region containing the *SIGLEC14*-*SIGLEC5* fusion in five minor allele carriers (two homozygous for *SIGLEC14* deletion and three heterozygous) [25]. Based on these sequencing data, relative to reference sequences, we found a 692 bp region of complete identity between *SIGLEC14* and *SIGLEC5*. Within this region, the deletion polymorphism sequence corresponds to *SIGLEC14* at the 5′ end, but *SIGLEC5* on the 3′ end, with respect to reference sequence data (Figure 1). Overall, this represents a 17 kb deletion.

### 3.3. SIGLEC14 CNV Is Not Fully Captured by rs1106476

**Figure 2:**
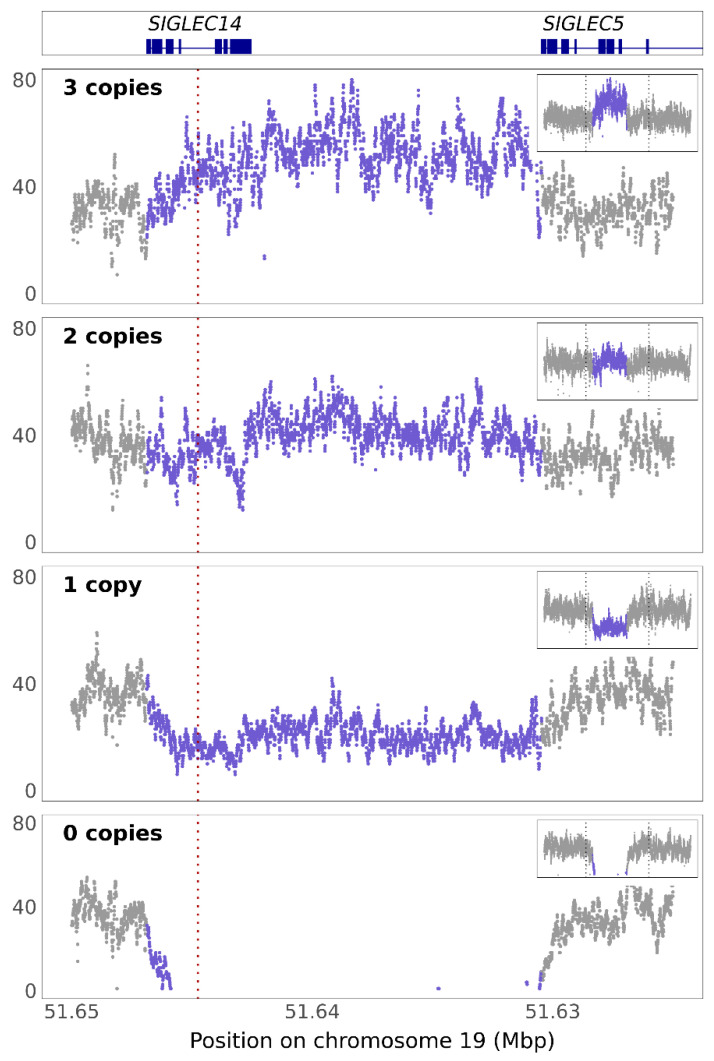
*SIGLEC14* CNVs detected in ADNI and ADSP cohorts. Read depth shown by chromosomal position of whole-genome sequencing in a representative example of each CNV detected. Exon/intron maps for *SIGLEC14* and *SIGLEC5* at figure top for reference. Purple: copy number variation. Inset: expanded view of locus. Red dotted line: location of copy number variation assay. The dotted line in the insets shows the boundaries of the full-size image.

As noted in Table 3, we found some individuals that had three copies of *SIGLEC14* as detected by the CNV assay. To validate these findings, we leveraged the ADNI and ADSP WGS datasets and compared read depth in the *SIGLEC14* locus with surrounding sequences (Figure 2). Both datasets contained individuals with *SIGLEC14* copy numbers ranging from 0–3. The presence of three copies of *SIGLEC14* was cross-validated between WGS data and CNV assay in three individuals. Further, the frequencies across populations are equivalent (Table 4; *p* = 6.76 × 10^−12^, χ^2^ = 69.30). Read depths for Caucasian, African American, and other populations are shown as Supplemental Figures S1–S3.

### 3.4. SIGLEC14 Is Expressed in Human Brain, and CNV Correlates with Gene Expression

**Figure 3:**
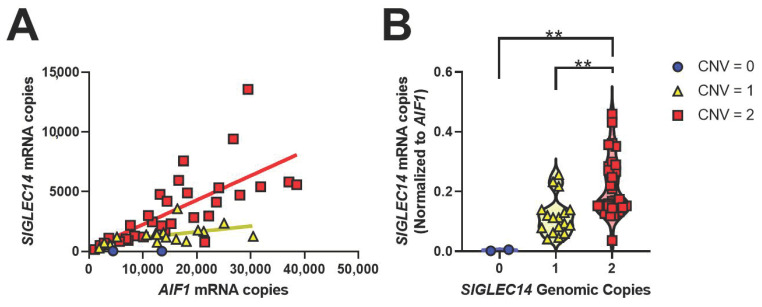
SIGLEC14 expression correlates with microglial gene AIF1 and SIGLEC14 CNV. (**A**) SIGLEC14 is expressed in microglia (*p* < 0.0001, F_1,48_ = 33.19, r^2^ = 0.409). (**B**) SIGLEC14 CNV strongly correlates with SIGLEC14 gene expression (*p* = 0.0002, F_2,47_ = 10.679), Tukey’s post-hoc multiple comparisons test. ** *p* < 0.01. We do not have statistical power to compare expression with CNV > 2, given its low MAF.

To test whether gene expression compensation may neutralize the effect of genomic *SIGLEC14* deletion, we quantified *SIGLEC14* expression relative to *SIGLEC14* gene copy number in cDNA prepared from human brain samples. Consistent with RNAseq studies that show *SIGLEC14* is expressed in microglia, *SIGLEC14* expression strongly correlated with expression of the microglial gene *AIF1* (*p* < 0.0001, r^2^ = 0.409, Figure 3A) [19,32]. When *SIGLEC14* expression is normalized to *AIF1* expression, *SIGLEC14* expression was dependent in a step-wise manner with *SIGLEC14* CNV (*p* = 0.0002, F_2,47_ = 10.679, Figure 3B). Strikingly, individuals with one copy of *SIGLEC14* have a mean *SIGLEC14* expression of 54.6% compared to individuals with two copies. We interpret this to mean that there is no compensatory increase in *SIGLEC14* expression in individuals heterozygous for *SIGLEC14* deletion.

### 3.5. SIGLEC14 Deletion Leads to Increased SIGLEC5 Expression

**Figure 4:**
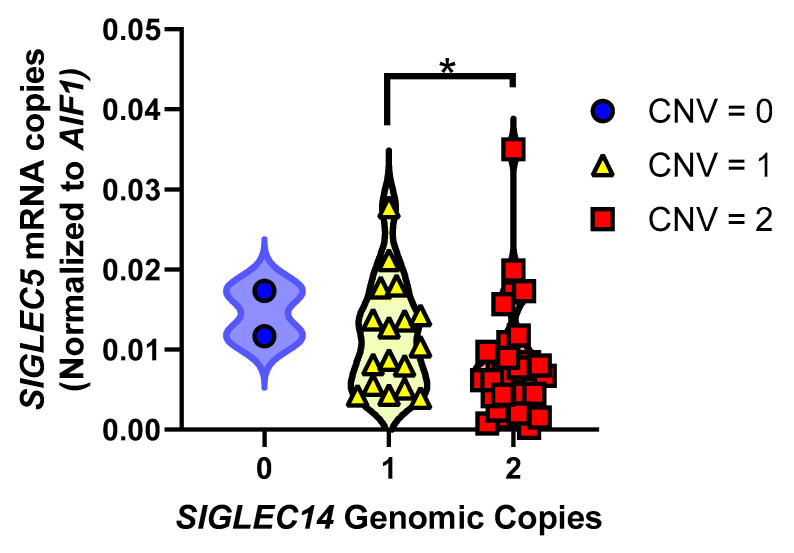
*SIGLEC5* expression inversely correlates with *SIGLEC14* CNV. *SIGLEC5* expression increases with fewer copies of *SIGLEC14*, presumably due to proximity to regulatory elements (*p* = 0.0220, F_2,46_ = 4.151), Tukey’s post-hoc multiple comparisons test. * *p* = 0.0389.

To test whether *SIGLEC5* expression changed with respect to *SIGLEC14* deletion, we quantified *SIGLEC5* expression relative to *SIGLEC14* CNV in these same brain samples. Since *SIGLEC5* does not have its own promoter and there are no H3K27 acetylation peaks between *SIGLEC14* and *SIGLEC5*, we hypothesized that an inverse relationship exists between *SIGLEC14* CNV and *SIGLEC5* expression, where a *SIGLEC14* deletion brings *SIGLEC5* closer to the promoter leading to increased transcription (Supplemental Figure S4) [33,34,35]. We found that *SIGLEC5* expression significantly increases with respect to *SIGLEC14* genomic deletions (Figure 4; *p* = 0.0220, F_2,46_ = 4.151).

## 4. Discussion

The primary finding of this paper is that pQTLs for ITIM and ITAM-containing proteins are overrepresented as being nominally significant for AD risk, suggesting that the ITIM and ITAM family of proteins may contribute to AD pathogenesis. This adds to the current body of work which supports the hypothesis that AD is mediated, at least in part, by immune cell dysfunction [1,4,5,36]. Indeed, transcriptomics and genomics studies have frequently identified genes predominantly expressed in microglia within the CNS as associated with AD risk [37,38,39,40,41]. Within a pQTL study, variants that affect the expression of the ITIM/ITAM family of genes—which govern immune cell activation state—are more commonly associated with AD risk than variants for genes, not in this family (Table 2). Although we hypothesized that variants that enhanced ITAM levels or decreased ITIM levels would be associated with reduced AD risk, this was not observed. This likely indicates that while some of these pQTLs may reflect increased functional signaling, others may involve alterations in splicing to generate soluble isoforms or may increase susceptibility to cleavage from the cell surface. Hence, an SNP that associates with increased plasma protein levels does not necessarily correlate with increased cell surface expression and signaling.

*SIGLEC14* was selected for further investigation based on its previously reported deletion polymorphism and close relationship to another AD-associated gene, *CD33* [2,25]. Since SNPs have previously been recognized as proxies for deletion of other genes [42,43,44], and SIGLEC14 deletion has been previously reported [25], we hypothesized that the strong pQTL signal from rs1106476 reported in Sun et al. [24] correlated with *SIGLEC14* deletion. Indeed, we found that rs1106476 is a proxy for *SIGLEC14* deletion and the minor allele count corresponds to the number of *SIGLEC14* deletions in 89% of cases in our dataset (Table 3).

This proxy variant does not, however, predict copy numbers greater than two. For instance, we observed four individuals with three copies of *SIGLEC14*; two of these individuals were homozygous minor for rs1106476 and two were heterozygous for rs1106476 (Table 3). Additional copy number variation is also present in the ADSP and ADNI sequencing projects (Figure 2). These CNVs are equivalent across populations in these datasets (Table 4, Supplemental Figures S1–S3). Based on these data and the recombination peak which spans from upstream of *SIGLEC14* through exon 8 of *SIGLEC5* (Supplemental Figure S5), we hypothesize that the additional copies integrate from a deletion event, though far less frequently than the deletion itself [45]. Across the 3095 individual WGS dataset in ADSP, we found *SIGLEC14* deletion has a minor allele frequency (MAF) of 0.2023, while insertion occurs at a MAF of only 0.0195, suggesting a 10-times lower rate of integration than deletion (Table 4).

In the brain, *SIGLEC14* is predominantly expressed in microglia, in keeping with its putative role as an immune receptor (Figure 3A). The *SIGLEC14* deletion polymorphism also strongly correlates with *SIGLEC14* gene expression (Figure 3B). Due to the low frequency of the additional copy integration, we do not have sufficient samples with which to correlate *SIGLEC14* expression to additional copy numbers, nor can we conclude whether additional *SIGLEC14* genomic copies are transcribed in frame and subsequently produce protein.

We also find that *SIGLEC14* deletion increases the expression of *SIGLEC5* (Figure 4). For individuals with at least one copy of *SIGLEC14*, the expression of *SIGLEC14* is substantially higher than *SIGLEC5*. Coupled with the lack of an independent promoter or H3K27 acetylation peaks between the two genes in GeneHancer or Encode, respectively, we infer that expression of both genes is governed by a common promoter proximal to *SIGLEC14*, that the integrity of this promoter is preserved after *SIGLEC14* deletion, and that *SIGLEC14* deletion results in an increase in *SIGLEC5* expression due to its closer proximity to this common element. The SIGLEC family of receptors bind sialic acids as ligands to initiate their signaling cascades, and sialylated proteins, as well as gangliosides, are abundant in amyloid plaques [46,47,48]. This decrease in expression of *SIGLEC14*, an ITAM-coupling protein, and concomitant increase in expression of *SIGLEC5*, an ITIM-containing protein, may lead to a dampened microglial activation state or proportion of activated microglia in deletion carriers. We speculate that decreased SIGLEC14 expression and increased SIGLEC5 expression may decrease the phagocytic capacity in AD. This is similar to the inverse relationship between *TREM2* and *CD33*, two well-known AD risk factors. Loss of the ITAM-containing TREM2 decreases phagocytic capacity, while loss of CD33 increases phagocytic capacity [11,13,49]. Since *TREM2*, which couples with DAP12, is critical for the transition of microglia into a full disease-associated phenotype, *SIGLEC14* may also contribute to this transition [50]. Future studies could investigate whether at the single-cell level *SIGLEC14* CNV affects disease-associated microglial induction.

Copy number variation may represent a relatively unexplored source of genetic variation in AD [51]. GWAS such as Jansen et al. rely on SNPs, which do not always capture the full range of variation [2]. Additionally, “camouflaged” genes such as *SIGLEC5* and *SIGLEC14* with high sequence identity due to gene duplication are challenging for WGS and WES technologies which rely on small fragments of DNA sequence, typically under 250 bp reads [51]. As such, variants which may have disease relevance and association may be overlooked with current methods. *SIGLEC14* is an example of one such possibly overlooked risk contributor in AD. *SIGLEC14* encodes an ITAM protein and signals through DAP12 similar to *TREM2*, and deletion of *SIGLEC14* is associated with increased AD risk, also similar to SNPs that reduce *TREM2* function [1,3,4,5]. Ligands for SIGLEC14, which include sialylated proteins, are commonly found within amyloid plaques similar to ligands for TREM2. We propose that the effect size and significance of association are masked through copy number variation not accounted for using the proxy SNP alone, i.e., loss of SIGLEC14 function likely increases risk, but the proxy SNP rs1106476 occasionally also marks the individuals with an extra *SIGLEC14* copy, thus reducing the power of rs1106476 association with AD. We thus conclude that *SIGLEC14* represents a potentially overlooked AD genetic risk factor due to complex genetics.

## Figures and Tables

**Table 1:** Genes that are nominally significant for AD association with strong pQTL signal.

Gene	SNP	P (pQTL)	β (pQTL)	P (AD)	β (AD)	N (AD)	ITIM/ ITAM
CD33	rs12459419	0 ^†^	−0.94	7.13 × 10^−9^	−0.01330	458,744	ITIM
FCGR3B	rs10919543	3.20 × 10^−67^	0.44	0.000317	0.00806	445,293	ITAM
LILRA5	rs759819	2.50 × 10^−111^	−0.54	0.00186	0.00717	454,216	ITAM
LILRB2	rs373032	7.60 × 10^−146^	−0.72	0.00227	0.00763	463,880	ITIM
SIGLEC9	rs2075803	0 ^†^	−1.23	0.00703	0.00576	466,252	ITIM
SIRPB1	rs3848788	1.20 × 10^−213^	0.75	0.00942	0.00582	458,092	ITAM
COLEC12	rs2846667	9.30 × 10^−12^	0.20	0.0177	0.00586	449,987	ITAM
FCRL1	rs4971155	6.30 × 10^−26^	−0.26	0.0197	−0.00520	403,829	ITAM
NCR1	rs2278428	1.10 × 10^−15^	−0.36	0.0249	0.00815	466,252	ITAM
SIGLEC14	rs1106476	0 ^†^	−1.19	0.0284	0.00736	458,063	ITAM
FCRL3	rs7528684	1.40 × 10^−112^	0.53	0.04	−0.00434	458,744	Both
MRC2	rs146385050	1.30 × 10^−11^	−0.22	0.041	−0.00612	396,686	ITAM
SLAMF6	rs11291564	2.60 × 10^−12^	0.20	0.042	−0.02450	17,477	ITAM

† The *p*-value in the analyzed summary statistics was reported as exactly 0. This does not impact our analysis, as our threshold was any cis-pQTL at *p* < 0.05.

**Table 2:** Overlap of pQTL and AD signals.

pQTLs	ITIM/ITAM (%)	Not ITIM/ITAM (%)	Total
AD *p* < 0.05	13 (28)	54 (10)	67
AD *p* > 0.05	34 (72)	488 (90)	522
**Total**	47 (100)	542 (100)	589

**Table 3:** Evaluation of rs1106476 as a proxy for *SIGLEC14* deletion.

*SIGLEC14* Copies	rs1106476 T/T	rs1106476 A/T	rs1106476 A/A	Total
0	0	1	1	2
1	6	13	0	19
2	39	0	0	39
3	2	2	0	4
Total	47	16	1	64

Blue = predicted correlation of *SIGLEC14* deletion vs. rs1106476. Each cell represents the number of DNA samples with the indicated *SIGLEC14* copy number and rs1106476 genotype.

**Table 4:** Summary of the *SIGLEC14* CNV in the 3095 sample ADSP WGS dataset.

*SIGLEC14* Copy Number	Caucasian	African American	Other	Total
**0**	24	74	44	142
**1**	304	348	316	968
**2**	692	522	652	1866
**3**	21	53	43	117
**4**	0	1	1	2
**Total**	1041	998	1056	3095
**Deletion MAF**	0.1691	0.2485	0.1913	0.2023
**Addition MAF**	0.0101	0.0276	0.0213	0.0195

MAF: Minor allele frequency.

## Data Availability

The Sun et al. proteomics dataset is available through the supplementary materials provided in the original publication, accessed on 30 January 2020 [24]. The Jansen et al. AD summary statistics are available through: https://ctg.cncr.nl/software/summary_statistics, accessed on 10 January 2019.

## Supplementary Materials

The following are available online at https://www.mdpi.com/article/10.3390/genes12071008/s1, Figure S1: Whole genome sequencing (WGS) read depth data from the Alzheimer’s Disease Sequencing Project (ASDP) in Caucasian population, Figure S2: WGS read depth data from the ASDP in African American population, Figure S3: WGS read depth data from the ASDP in all other populations, Figure S4: The *SIGLEC14* locus contains no H3K27Ac peaks nor regulatory elements between *SIGLEC14* and *SIGLEC5*. Expression of *SIGLEC14* is approximately ten times higher than *SIGLEC5* in individuals with both copies of *SIGLEC14*, while *SIGLEC5* expression is higher in individuals lacking *SIGLEC14* copies, in keeping with a common promoter or enhancer governing the single locus, Figure S5: *SIGLEC5* and *SIGLEC14* share a broad recombination peak (gray line). Note that, since *SIGLEC14* and *SIGLEC5* are on the minus strand, these genes appear inverted in this figure and read right-to-left, Table S1: List of ITIM/ITAM genes and their aliases.
