# Supplementary material for: Analysis of Genetic Variants Associated with Levels of Immune Modulating Proteins for Impact on Alzheimer’s Disease Risk Reveal a Potential Role for SIGLEC14
Source: Genes (Basel). 2021 Jun 30;12(7):1008. doi: 10.3390/genes12071008 (PMC8303736; doi:10.3390/genes12071008)
Supplement: Supplementary file 1 [file genes-12-01008-s001.zip › Supplemental Information.pdf]

Supplementary Material

Genetic variants associated with levels of immune modulating proteins are overrepresented as Alzheimer's Disease risk factors

Benjamin C. Shaw, MS[1,2], Yuriko Katsumata, PhD[2,3], James Simpson, MS[1,2], David Fardo, PhD[2,3], and Steven Estus, PhD[1,2*]

[1]*Department of Physiology, University of Kentucky*

[2]*Sanders-Brown Center on Aging, University of Kentucky*

[3]*Department of Biostatistics, University of Kentucky*

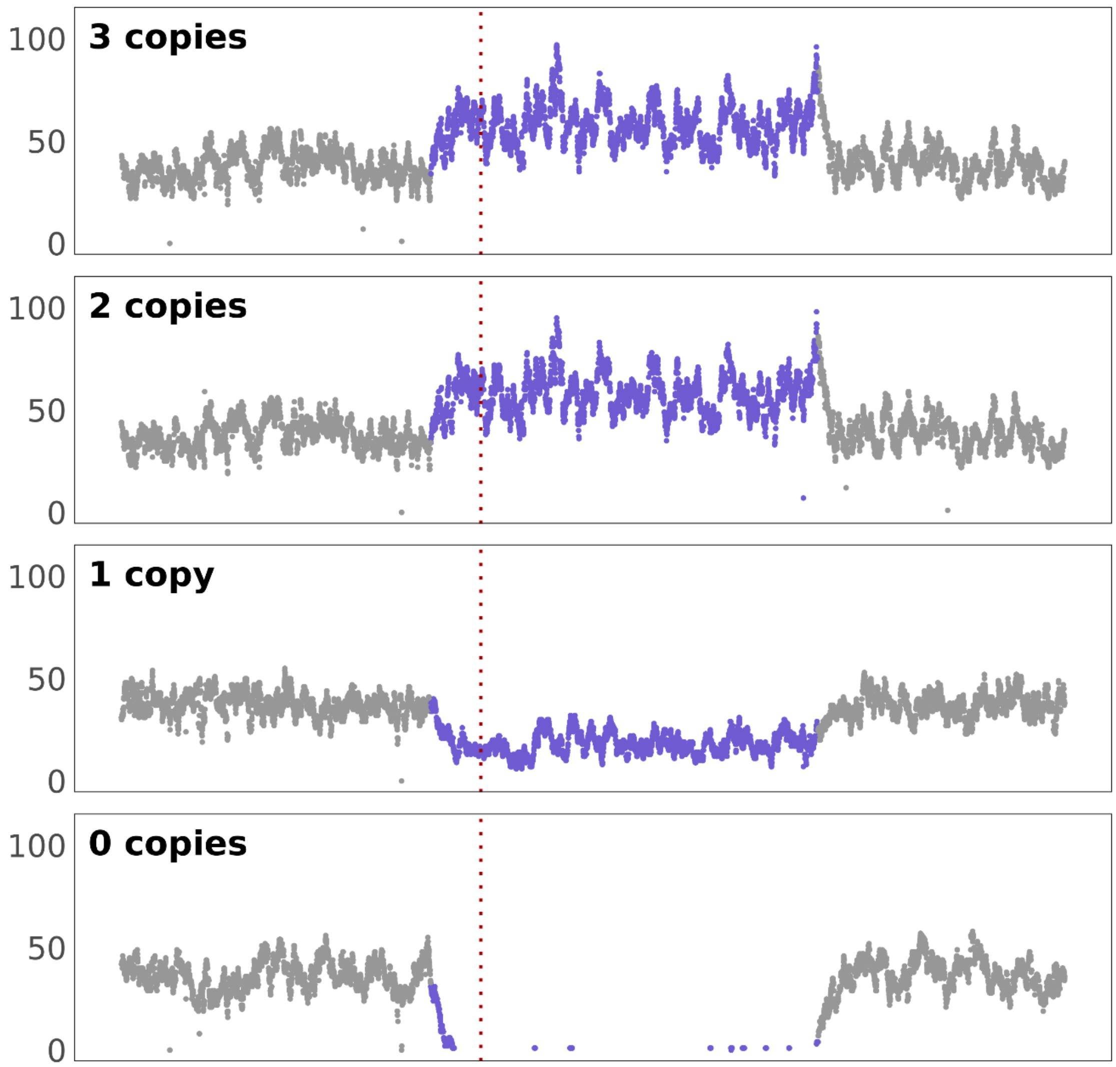

Figure S1: Whole genome sequencing (WGS) read depth data from the Alzheimer's Disease Sequencing Project (ASDP) in Caucasian population (n = 1041) reveals copy number variation (CNV) in *SIGLEC14*, including deletions and duplications.

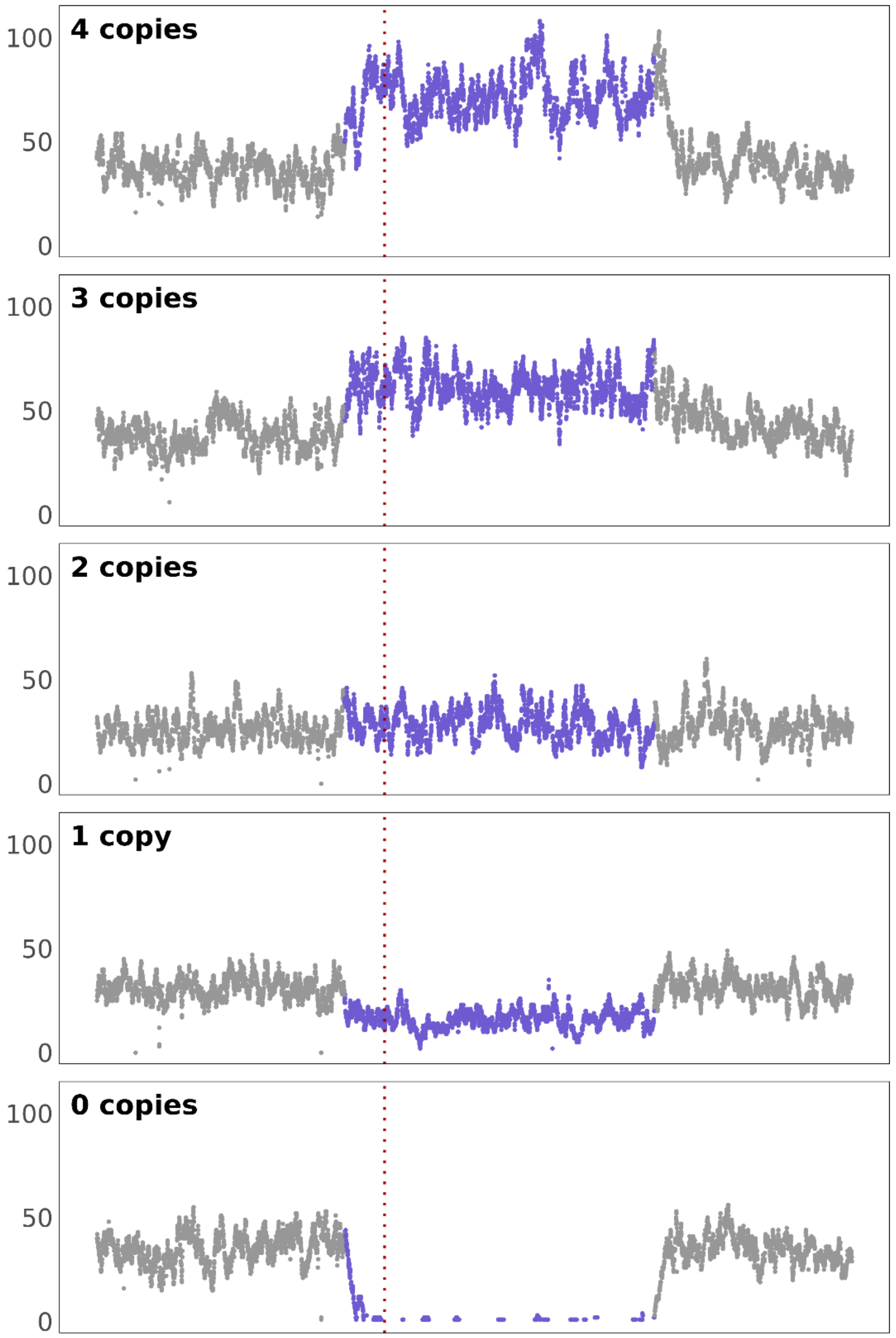

Figure S2: WGS read depth data from the ASDP in African American population (n = 998) reveals CNV in *SIGLEC14*, including deletions and duplications. Especially notable is the 4-allele carrier. Frequencies described in Table 4.

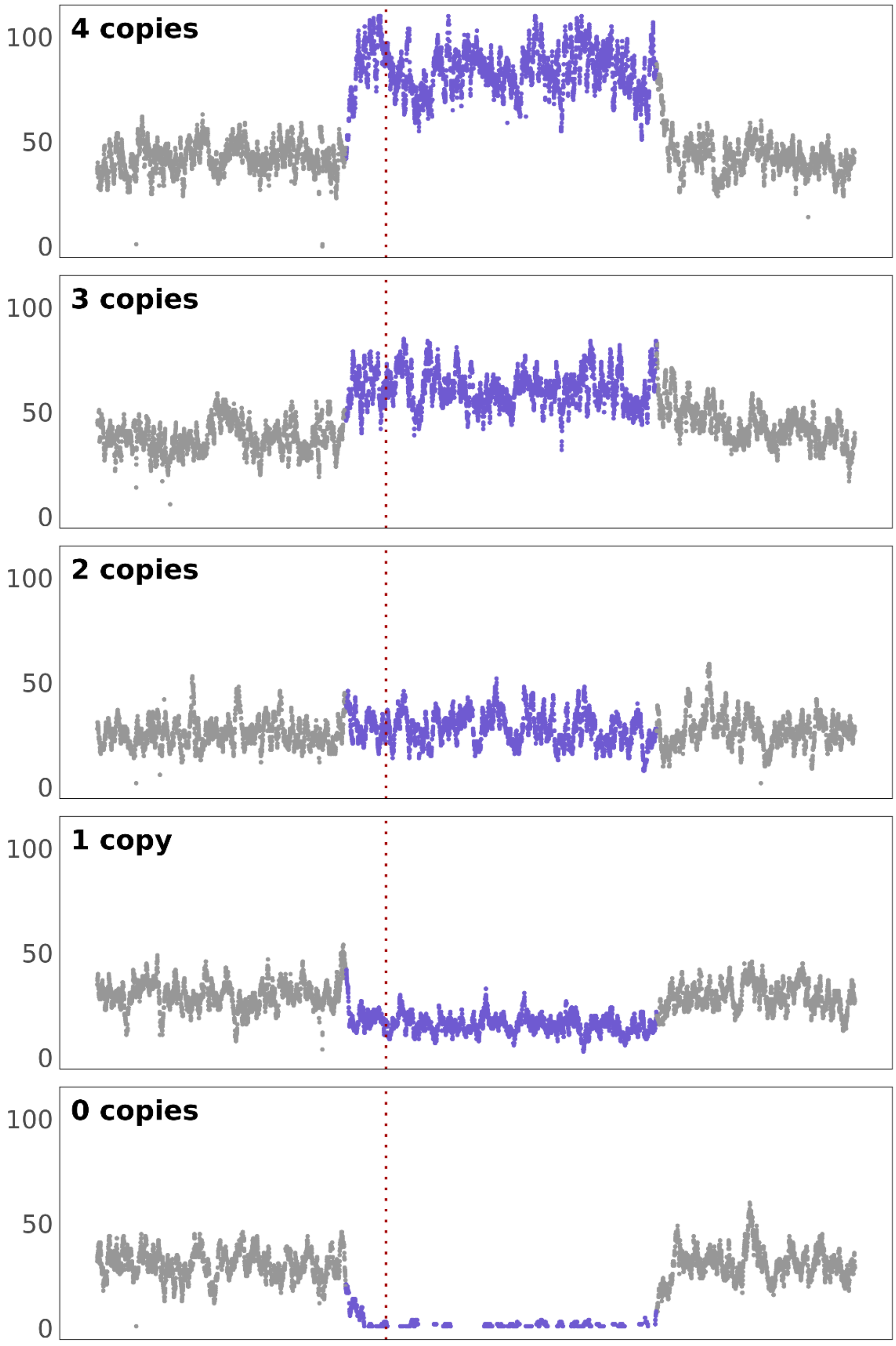

Figure S3: WGS read depth data from the ASDP in all other populations (n = 1056) reveals CNV in *SIGLEC14*, including deletions and duplications. Especially notable is the 4-allele carrier. Frequencies described in Table 4.

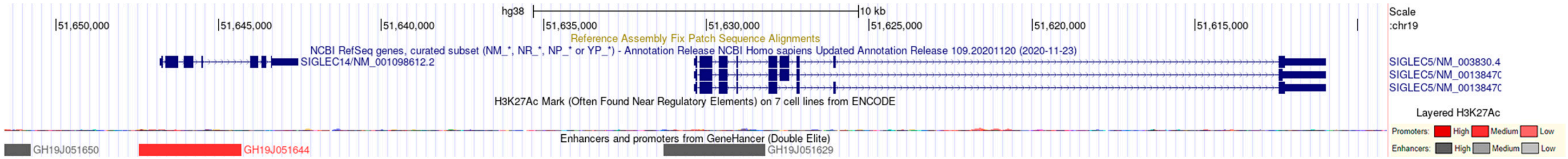

Figure S4. The *SIGLEC14* locus contains no H3K27Ac peaks nor regulatory elements between *SIGLEC14* and *SIGLEC5*. Expression of *SIGLEC14* is approximately ten times higher than *SIGLEC5* in individuals with both copies of *SIGLEC14*, while *SIGLEC5* expression is higher in individuals lacking *SIGLEC14* copies, in keeping with a common promoter or enhancer governing the single locus.

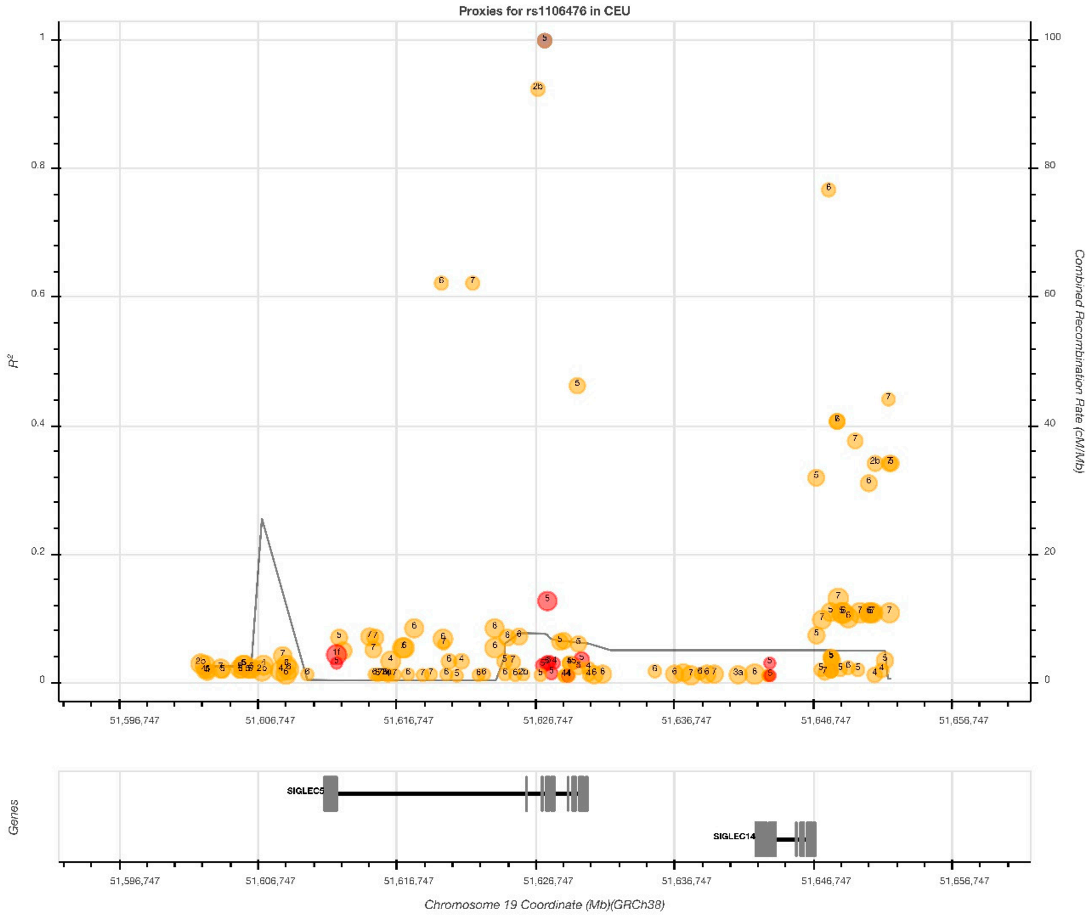

Figure S5. *SIGLEC5* and *SIGLEC14* share a broad recombination peak (gray line). Note that, since *SIGLEC14* and *SIGLEC5* are on the minus strand, these genes appear inverted in this figure and read right-to-left.

Table S1: Provided as a .csv file. Contains all 187 ITIM/ITAM genes used in the analysis, including aliases.
